# Ameliorative effects of eosinophil deficiency on immune response, endoplasmic reticulum stress, apoptosis, and autophagy in fungus-induced allergic lung inflammation

**DOI:** 10.1186/s12931-021-01770-4

**Published:** 2021-06-07

**Authors:** Sijiao Wang, Zhilong Jiang, Liyang Li, Jun Zhang, Cuiping Zhang, Changzhou Shao

**Affiliations:** 1 Department of Pulmonary Medicine, Shanghai Respiratory Research Institute, Zhongshan Hospital, Fudan University, Shanghai, 200032 China; 2 Department of Pulmonary Medicine, Xiamen Branch, Zhongshan Hospital, Fudan University, Xiamen, 361015 China

**Keywords:** *Aspergillus fumigatus*, Allergic lung disease, Eosinophil deficiency, Immune response, Inflammation

## Abstract

**Background:**

Respiratory fungal exposure is known to be associated with various allergic pulmonary disorders. Eosinophils have been implicated in tissue homeostasis of allergic inflammation as both destructive effector cells and immune regulators. What contributions eosinophils have in *Aspergillus fumigatus* (Af)-induced allergic lung inflammation is worthy of investigating.

**Methods:**

We established the Af-exposed animal asthmatic model using eosinophil-deficient mice, ∆dblGATA1 mice. Airway inflammation was assessed by histopathological examination and total cell count of bronchoalveolar lavage fluid (BALF). The protein level in BALF and lung mRNA level of type 2 cytokines IL-4, IL-5, and IL-13 were detected by ELISA and qRT-PCR. We further studied the involvement of endoplasmic reticulum (ER) stress, apoptosis, and autophagy by western blots, qRT-PCR, immunofluorescence, TUNEL, or immunohistochemistry. RNA-Seq analysis was utilized to analyze the whole transcriptome of Af-exposed ∆dblGATA1 mice.

**Results:**

Hematoxylin and eosin (HE) staining and periodic acid–Schiff staining (PAS) showed that airway inflammation and mucus production were alleviated in Af-challenged ∆dblGATA1 mice compared with wild-type controls. The protein and mRNA expressions of IL-4, IL-5, and IL-13 were reduced in the BALF and lung tissues in Af-exposed ∆dblGATA1 mice. The results demonstrated that the significantly increased ER stress markers (GRP78 and CHOP) and apoptosis executioner caspase proteases (cleaved caspase-3 and cleaved caspase-7) in Af-exposed wild-type mice were all downregulated remarkably in the lungs of ∆dblGATA1 mice with Af challenge. In addition, the lung autophagy in Af-exposed ∆dblGATA1 mice was found elevated partially, manifesting as higher expression of LC3-II/LC3-I and beclin1, lower p62, and downregulated Akt/mTOR pathway compared with Af-exposed wild-type mice. Additionally, lung RNA-seq analysis of Af-exposed ∆dblGATA1 mice showed that biological processes about chemotaxis of lymphocytes, neutrophils, or eosinophils were enriched but without statistical significance.

**Conclusions:**

In summary, eosinophils play an essential role in the pathogenesis of Af-exposed allergic lung inflammation, whose deficiency may have relation to the attenuation of type 2 immune response, alleviation of ER stress and apoptosis, and increase of autophagy. These findings suggest that anti-eosinophils therapy may provide a promising direction for fungal-induced allergic pulmonary diseases.

## Introduction

*Aspergillus fumigatus* (Af) is an ubiquitous fungus that can produce small enough airborne conidia to reach the lower airways to elicit a destructive response [1]. Af exposure could induce a series of allergic pulmonary disorders, such as allergic bronchopulmonary aspergillosis (ABPA) and severe asthma with fungal sensitization (SAFS) [2]. It was estimated that ABPA affected approximately 4.8 million people globally [3], and over 70% of patients with severe asthma were sensitized to at least one fungus [4]. However, for patients with fungus exposure, conventional treatment using inhaled corticosteroids seems inadequate, and combination therapy such as systemic corticosteroid and antifungal agents has been suggested [4, 5]. Therefore, it was essential to explore the potential mechanisms for Af-induced allergic lung inflammation to offer promising therapeutic options.

Eosinophils, terminally multi-functional leukocytes, have been implicated in allergic airway diseases as both end-stage destructive effector cells and immune regulators [6]. Eosinophils have been shown to induce airway damage and mucin production by secreting toxic granule proteins [7, 8], and participate in immunoregulation such as processing antigens [9], inducing the accumulation of dendritic cells into the lung [10], producing chemokines to recruit T cells, and secreting Th2-polarizing cytokines [7, 8]. However, whether eosinophil deficiency could impact the cellular processes such as endoplasmic reticulum (ER) stress, apoptosis, and autophagy in allergic pulmonary disease remains to be elucidated.

Endoplasmic reticulum is the largest organelle responsible for protein synthesis, assembly, transport, and degradation, where the accumulation of misfolded or unfolded proteins always trigger adaptive mechanisms unfolded protein response (UPR) and ER stress if UPR was uncontrolled [11]. It was shown that ER stress inhibitors could alleviate allergic inflammation induced by ovalbumin (OVA) or fungal allergen [12–14]. Also, prolonged activation of ER stress could induce apoptosis [15], and epithelial apoptosis inhibition could protect against airway damage in OVA, house dust mite (HDM), or *Alternaria Alternata* allergen challenged mice [16–18]. Besides, as an essential and highly conserved mechanism for maintaining cellular homeostasis [19], macroautophagy, one of the most prevalent form of autophagy, displayed a controversial role in asthmatic murine models because of its protective [20] or detrimental [21, 22] effects. ER stress and apoptosis in response to inflammation promote airway epithelial damage, while autophagy acted as a double-edged sword under inflammatory condition within airway [23]. Previous studies have shown that eosinophil deficiency ameliorated allergic airway inflammation [7, 8, 24]. What happens to ER stress, apoptosis and autophagy under eosinophil deficiency deserves to be explored.

This study elucidated the ameliorative effects of eosinophil deficiency in the Af-induced allergic lung inflammation utilizing ∆dblGATA1 mice. We observed alleviated airway inflammation, decreased mucus secretion, and reduced type 2 cytokines when eosinophils were deficient in Af-induced allergic lung inflammation. We also found activated ER stress, increased lung epithelial apoptosis, and reduced autophagy in Af-induced allergic lung inflammation, which were prevented partially in Af-exposed ∆dblGATA1 mice. Taken together, in light of the potential associations between eosinophil deficiency and immune response, stress response, and cellular homeostasis, our findings indicate that eosinophils play a critical role in Af-induced allergic pulmonary disorders.

## Materials and methods

### Animals

∆dblGATA1 mice (BALB/c background) were purchased from the Jackson laboratory. Female wild-type (WT) BALB/c mice (6–8 weeks) were obtained from SLAC Laboratory Animals (Shanghai, China). All mice were housed under specific pathogen-free conditions and maintained on a 12 h light–dark rhythm in the Animal Center of Zhongshan Hospital, Fudan University. Four groups (4–6 mice/group) were designed and the experiment was repeated three times. This study was approved by the Animal Care Committee of Fudan University Zhongshan Hospital (ID:2019-020).

### Animal experiments protocols

**Fig. 1:**
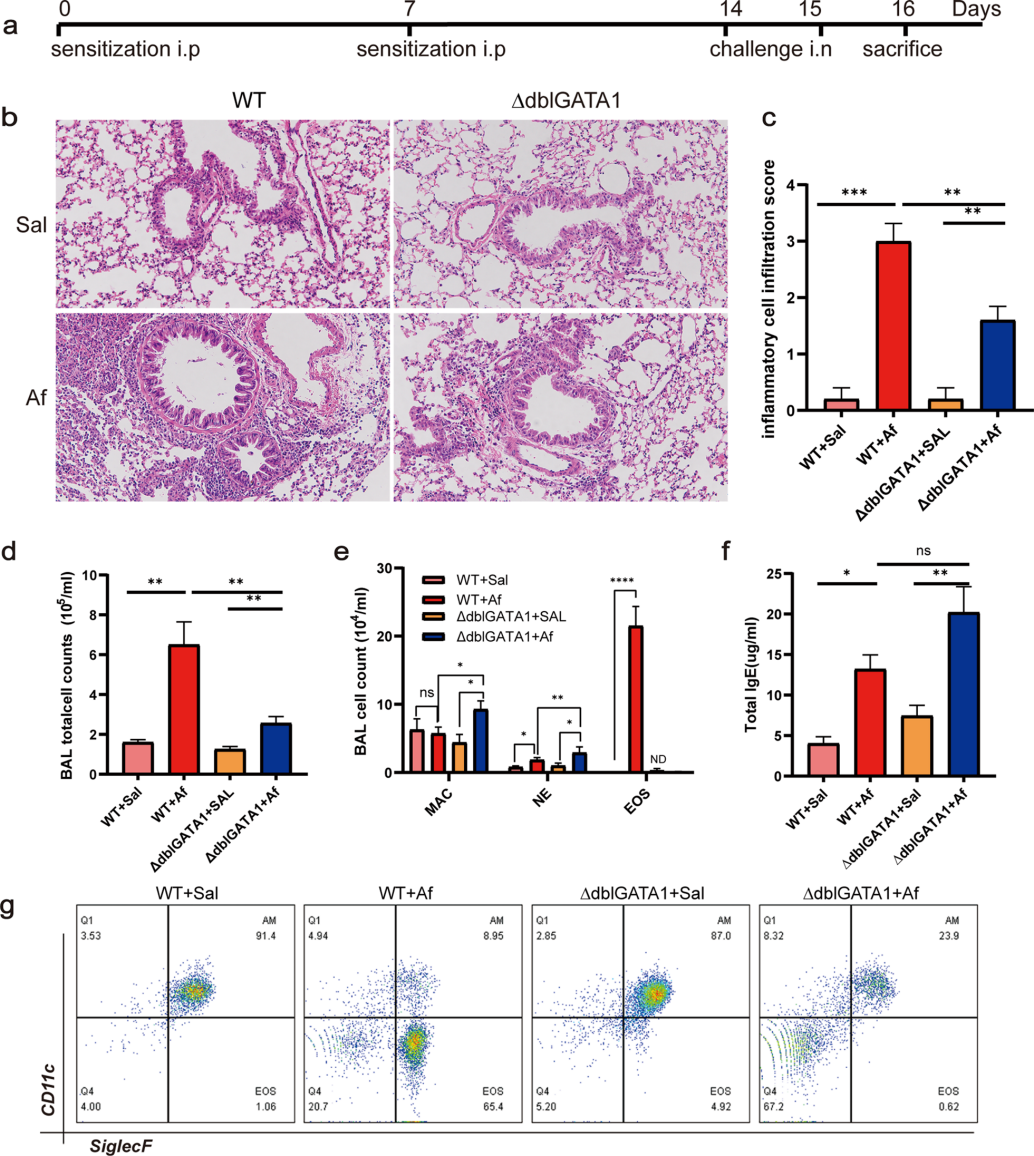
Eosinophil deficiency alleviated Af-induced allergic airway inflammation. **a** Protocol of experiments for sensitization and challenge in mice. **b**, **c** Airway inflammation was determined by H&E staining (magnification, 200×) and scoring the extent of inflammation in the four groups. **d**, **e** The count of total cells (**d**) and eosinophils (EOS), neutrophils (NE), and macrophage (MAC) in BALF (**e**). **f** The total serum IgE measured by ELISA. **g** Flow cytometric dot plots of eosinophils and macrophages gated on CD45^+^ cells in BALF. Data are mean ± SEM. (n = 4–6) and the experiment was repeated three times. *p < 0.05, **p < 0.01, ***p < 0.001, ****p < 0.0001, and ns = no significance

According to the protocol as previously described [13, 25], briefly, as shown in Fig. 1a mice were sensitized intraperitoneally (i.p) with *Aspergillus fumigatus* extracts (Af, 20 μg per mouse; Greer Laboratories, Lenoir, North Carolina, USA) emulsified with aluminium (Imject Alum; Thermo Fisher Scientific, New York, USA) on days 0 and day 7, followed by intranasal inoculation (i.n) with 25 μg Af extracts on day 14 and day 15. The control mice were sensitized and challenged with saline. Twenty-four hours after the last challenge, all mice were sacrificed for lung tissue and blood. Bronchoalveolar lavage was performed twice with 1 ml of PBS via a tracheal catheter (80–90% recovery rate).

### Analysis of bronchoalveolar lavage fluid (BALF)

BALF was centrifuged at 300*g*, 5 min. The supernatant was stored at − 80 °C until analysis. Cell sediments were resuspended with PBS and stained with APC/Cyanine7 anti-CD45, PE/Cyanine7 Ly6G, APC-CD11c, and PE-Siglec-F, which were purchased from BioLegend (San Diego, CA, USA) or eBioscience (San Diego, CA, USA). Eosinophils were identified as CD45^+^ Ly6G^−^ CD11c^−^ Siglec-F^+^, neutrophils as CD45^+^ Ly6G^+^, and alveolar macrophages as CD45^+^ Ly6G^−^ CD11c^+^Siglec-F^+^. Hemocytometer was used to count total cells in BAL.

### Total IgE and cytokines measurement

Serum IgE level was measured by ELISA (Invitrogen, Thermo Fisher Scientific, Inc). IL-4, IL-5, and IL-13 in BALF were measured by ELISA DuoSet kit (R&D system) according to the manufacturers’ instructions.

### RNA extraction and quantitative PCR analysis

Total RNA of lung tissue was extracted by Trizol reagents and then was reverse transcribed to cDNA using Reverse transcript RNA kit (Toyobo, Shiga, Japan). The specific transcripts were quantified by real time-PCR using SYBR (Toyobo, Shiga, Japan), and analyzed with ABI 7500 RT-PCR system (Applied Biosystems, Foster, CA, United States). The primers of genes were synthesized by Sangon Biotech, and their sequences were shown in Table 1. RNA relative expression levels were normalized to β-actin mRNA level.

**Table 1:** Primers used in the study

Genes	Forward	Reverse
IL-4	GGTCTCAACCCCCAGCTAGT	GCCGATGATCTCTCTCAAGTGAT
IL-13	CCTGGCTCTTGCTTGCCTT	GGTCTTGTGTGATGTTGCTCA
IL-5	GCAATGAGACGATGAGGCTTC	GCCCCTGAAAGATTTCTCCAATG
Muc5ac	GGACTTCAATATCCAGCTACGC	CAGCTCAACAACTAGGCCATC
CHOP	TGGAAGCCTGGTATGAGGAT	CAGGGTCAAGAGTAGTGAAGGT
GRP78	ACTTGGGGACCACCTATTCCT	ATCGCCAATCAGACGCTCC
ATF4	AAGGAGGAAGACACTCCCTCT	CAGGTGGGTCATAAGGTTTGG

### Histopathology

Lungs were harvested, and the left lower lobe was fixed in 4% formalin, embedded in paraffin, and cut into 5-μm sections. Sections were dewaxed and rehydrated for hematoxylin and eosin (HE) and periodic acid schiff (PAS). The degree of airway inflammation was evaluated base on a scale of 0–4 (0, none; 1, mild; 2, moderate; 3, marked; and 4, severe) in a double‑blind manner [26].

### Western blot analysis

Lung tissues were lysed in RIPA buffer containing protease inhibitor and phosphatase inhibitor cocktail. The proteins were obtained from the supernatant after centrifugation. Protein concentrations were measured with the BCA Assay Kit (Beyotime, Shanghai, China). Lyses were resolved on sodium dodecyl sulfate-polyacrylamide gel electrophoresis (SDS-PAGE) and transferred to polyvinylidene fluoride (PVDF) membranes with 0.22 µm pore-size. The membranes were blocked for 1 h in 5% non-fat milk and incubated overnight at 4 ℃ with primary antibodies against CHOP, GRP78, cleaved caspase-3, cleaved caspase-7, phosphorylated (p)-mTOR-Ser2448 (Cell Signaling Technology, Boston, USA), LC3A/B, p62 (Abcam, Cambridge, United Kingdom), p-Akt1-ser 473, beclin1 (ABclonal, Wuhan, China), and β tubulin (Proteintech, Wuhan, China) at 1:1000 in primary antibody diluent, washed three times with Tris-buffered saline containing 0.1% Tween 20, and then incubated with horseradish peroxidase (HRP)-conjugated secondary antibody for 1 h. The density was detected by chemiluminescence. Densitometry was performed using Image J 2.0.

### Immunofluorescence

The lung sections were first deparaffinized according to the standard protocol. After antigen retrieval, slides were permeabilized, blocked with fetal bovine serum, washed three times with PBS (5 min each), and then incubated overnight at 4 ℃ with primary antibody against GRP78 antibody (1:200; Cell Signaling Technology). Next, followed by a PBS wash three times (5 min each), the slides were incubated with secondary antibody (1:500) for 1 h at room temperature. Lastly, the slides were then stained with DAPI and mounted after a PBS wash again. The tissue was visualized and photographed using Nikon Eclipse fluorescent microscope. The mean fluorescence intensity was calculated with the Image J 2.0.

### Immunohistochemistry

After deparaffinization and gradient dehydration, the endogenous peroxidase activity of slides was blocked by treating with 3% hydrogen peroxide for 10 min followed by a PBS wash three times (5 min each). The sections were incubated with a p62 antibody (1:200; Cell Signaling Technology) overnight at 4 ℃. The sections were then washed, incubated with HRP-conjugated secondary antibody for 1 h at room temperature. The secondary antibody was detected with DAB Substrate Kit, and slides were then counterstained with hematoxylin and mounted.

### Terminal deoxynucleotidyl transferase-mediated nick end labeling assay (TUNEL)

The apoptotic cells were detected in situ with TUNEL staining according to the manufacturer’s instructions (Servicebio, Wuhan, China). Images were captured by (Nikon, Tokyo, Japan). Apoptosis was quantified by determining the percentage of positively stained cells in DAPI positive cells in three random fields at 20× magnification.

### RNA-Seq analysis

Total RNA was extracted from lung tissues using Trizol according to instruction. Followed by qualitative and quantitative tests, RNA was sequenced using the BGISEQ-500 platform. RSEM was used to quantified the Genes expression level (FPKM). Genes differentially expressed between the two samples were identified by a strict algorithm (DEGseq). Gene Ontology (GO) and pathway annotation and enrichment analyses were based on the Gene Ontology Database (http://www.geneontology.org/) and the KEGG pathway database (http://www.genome.jp/kegg/), respectively.

### Statistical analysis

Data were expressed as the mean ± SEM and analyzed with GraphPad Prism version 8.0 software (GraphPad Software Inc, San Diego, CA, USA). Statistical comparisons were performed using Student’ s *t* test between two groups with a probability value p ≤ 0.05 considered statistically.

## Results

### Af-induced allergic airway inflammation was alleviated in ∆dblGATA1 mice

Histological examination showed significant infiltration of airway inflammatory cells in the peribronchial and perivascular area in Af-exposed wild-type mice, while it was attenuated in Af-exposed ∆dblGATA1 mice (Fig. 1b, c). A significantly decreased count of total BAL cells was observed in the Af-challenged ∆dblGATA1 mice (Fig. 1d). Moreover, eosinophils distinguished by flow cytometry (CD45^+^Ly6G^−^CD11c^−^Siglec-F^+^) were the dominant inflammatory cells in BALF of Af exposed wild-type mice, while the significant accumulation of neutrophils and almost undetected eosinophils were found in the ∆dblGATA1 with Af challenge (Fig. 1e, g). The total IgE level in serum was significantly higher in mice with Af challenge than those with saline no matter in wild-type or ΔdblGATA1 mice (Fig. 1f). These results were consistent with previous observations [8, 24], suggesting that eosinophil deficiency may reduce the allergic airway inflammation in Af-challenged mice.

### Th2 immune response was attenuated in Af-exposed ∆dblGATA1 mice compared with that of wild-type

**Fig. 2:**
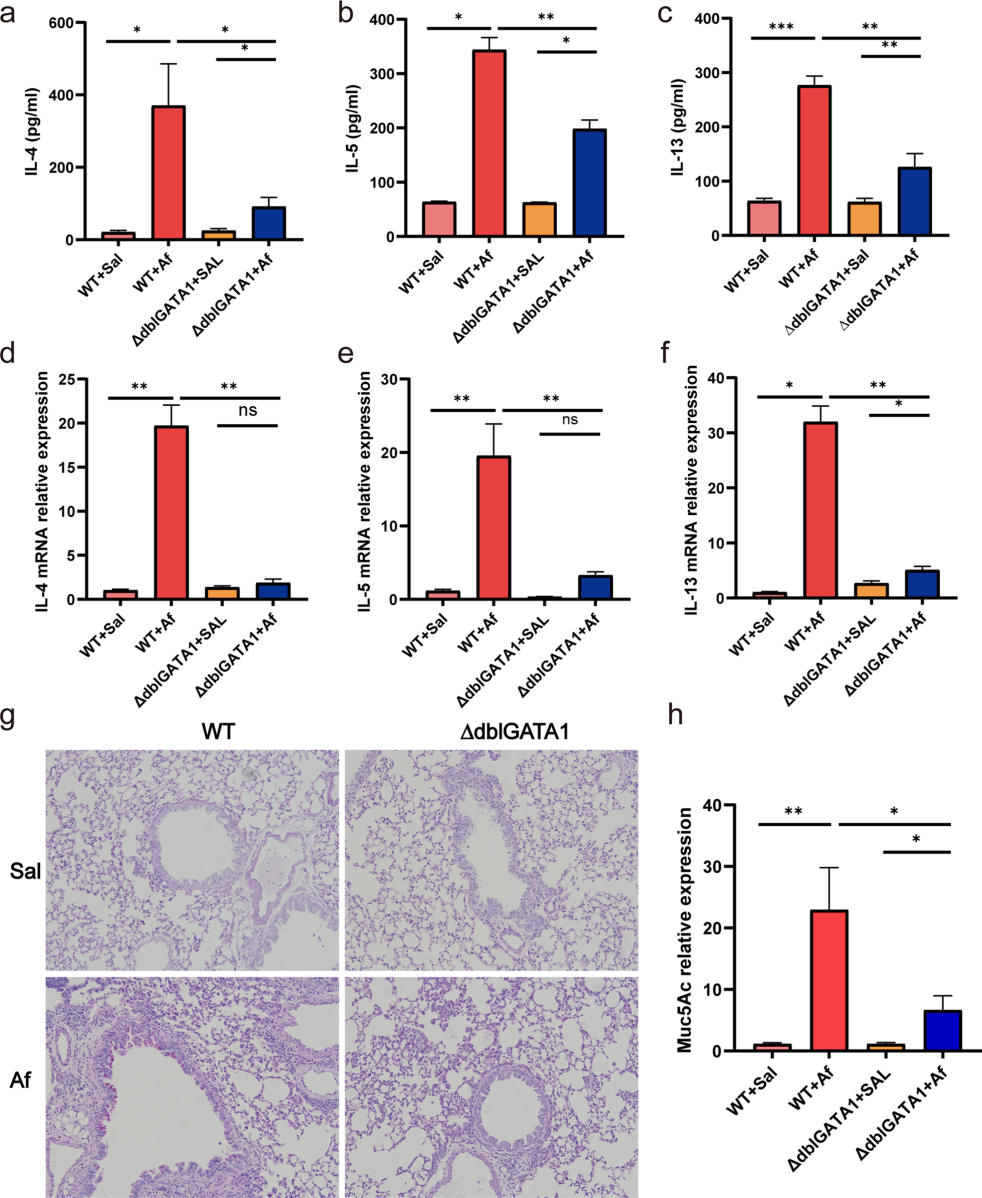
Eosinophil deficiency decreased Af-induced production of Th2 cytokines and mucin hyperproduction. **a**–**c** The protein levels of IL-4 (**a**), IL-5 (**b**), and IL-13 (**c**) in BALF quantified by ELISAs. **d**–**f** The mRNA expressions of IL-4 (**d**), IL-5 (**e**), and IL-13 (**f**) quantified by quantitative PCR. **g** Periodic acid Schiff staining showing mucus production (magnification, 200×). **h** mRNA level of lung mucin 5AC (Muc5AC). Data are mean ± SEM. (n = 4–6). Values for qPCR were normalized to β-actin. *p < 0.05, **p < 0.01, ***p < 0.001, and ns = no significance

Previous studies have shown that eosinophils are required to recruit effector T cells to evoke Th2 immune response in the lung [7]. We examined the gene level of type 2 cytokines in the lungs and their protein level in the BALF. As shown in Fig. 2a–c, after Af sensitization and challenge, the protein expressions of lung IL-4, IL-5, and IL-13 were significantly upregulated in wild-type mice and decreased in ∆dblGATA1 mice (p < 0.01). Consistently, the relative mRNA levels of IL-4, IL-5, and IL-13 in BALF were reduced in Af-exposed ∆dblGATA1 mice compared to wild-type controls (Fig. 2d–f, p < 0.01). Moreover, ∆dblGATA1 mice had lower mucin hyperproduction induced by Af than the wild-type as shown by PAS (Fig. 2g). Similarly, the gene level of lung Muc5AC was lower in the Af-exposed ∆dblGATA1 mice than that of wild-type, although it was increased in contrast to saline controls (Fig. 2h, p < 0.01). These data indicated that eosinophil deficiency has the potential to modulate Th2 immune response and mucus production in Af-challenged allergic lung inflammation [7, 10, 24].

### Af-exposed ∆dblGATA1 mice exhibited decreased lung ER stress level in contrast to wild type

**Fig. 3:**
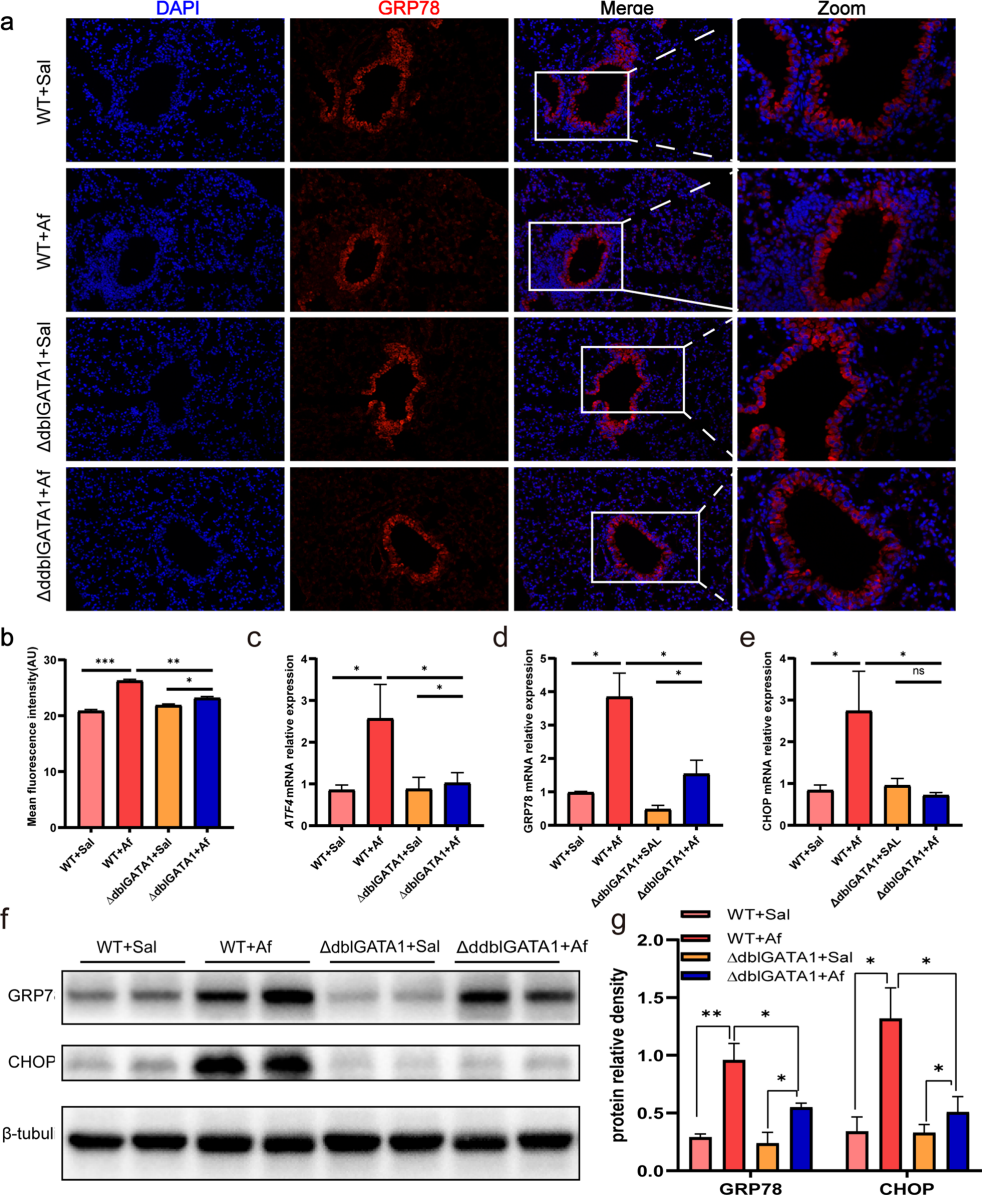
Eosinophils deficiency alleviated ER stress in the lungs of Af-exposed mice. **a**, **b** Representative immunofluorescence photomicrographs of lung tissues showing the expression and localization of GRP78 (magnification, 200×) and the quantified mean fluorescence intensity. Data are mean ± SEM (n = 3–5). **c**–**e** mRNA levels of ATF4, GRP78, and CHOP in the lung tissues. **f**, **g** The proteins levels of GRP78 and CHOP in the lungs were analyzed by Western blots. Values for qPCR were normalized to β-actin. Data are mean ± SEM. (n = 4–5). *p < 0.05, **p < 0.01, and ns = no significance

It had suggested that ER stress played a crucial role in Af-induced allergic lung inflammation [13]. To evaluate whether ER stress was affected in the context of eosinophil deficiency, we tested the ER stress markers, glucose-regulated protein 78 (GRP78) and C/EBP-homologous protein (CHOP) and one of the UPR-related markers, activating transcription factor (ATF4) [13]. Immunofluorescence showed that GRP78 was highly expressed at bronchial epithelial cells, and semi-quantitative analysis showed that expression of GRP78 was decreased in Af-exposed ∆dblGATA1 mice relative to Af-exposed wild-type (Fig. 3a, b, p < 0.01). Furthermore, GRP78, CHOP and ATF4 at the lung gene level were all upregulated in Af-exposed wild-type mice relative to controls, which were reduced in ∆dblGATA1 mice (Fig. 3c–e). Similarly, protein levels of GRP78 and CHOP were substantially reduced in ∆dblGATA1 mice compared with the wild-type after Af challenged, whereas they were higher than those of the saline controls (Fig. 3f, g).

### Af-exposed ∆dblGATA1 mice demonstrated improved cell apoptosis of lungs relative to wild type

**Fig. 4:**
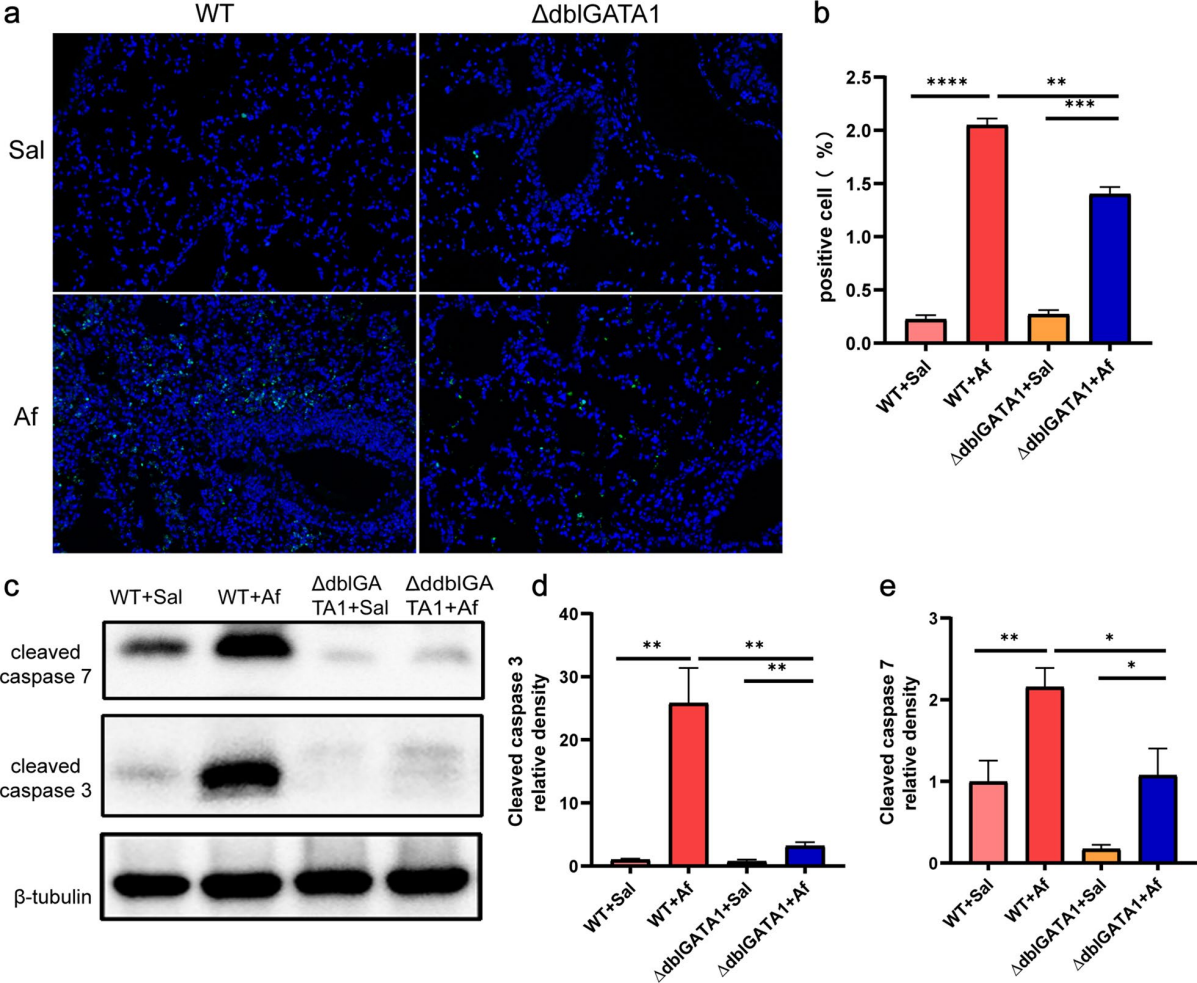
Eosinophils deficiency downregulated apoptosis in the lungs of Af-exposed mice. **a**, **b** Representative images of TUNEL assay (magnification: 200×) and quantification of TUNEL-positive cells in the lungs. **c**–**e** The proteins levels of cleaved caspase-7 and cleaved caspase-3 in the lungs measured by Western blots. Data are mean ± SEM. (n = 4–5) and the experiment was repeated three times. *p < 0.05, **p < 0.01, ***p < 0.001, ****p < 0.0001, and ns = no significance

To determine the effect of eosinophil deficiency on cell stress, we used TUNEL assays to detect cell apoptosis. Increased apoptosis in the lungs was observed in both wild-type mice and ∆dblGATA1 mice underwent Af challenge, and ∆dblGATA1 mice displayed a relatively milder change of apoptosis than wild-type mice (Fig. 4a, b). Western blots showed that cleaved caspase-3 and cleaved caspase-7 were significantly increased in the Af exposed wild-type mice (p < 0.01), which were reduced evidently in Af-exposed ∆dblGATA1 mice (Fig. 4c, e). Thus, these findings showed that eosinophils might be critical for the development of apoptosis in Af-challenged allergic lung inflammation.

### The lung autophagy was reduced in Af-exposed mice and was restored partially in Af-exposed ∆dblGATA1 mice

**Fig. 5:**
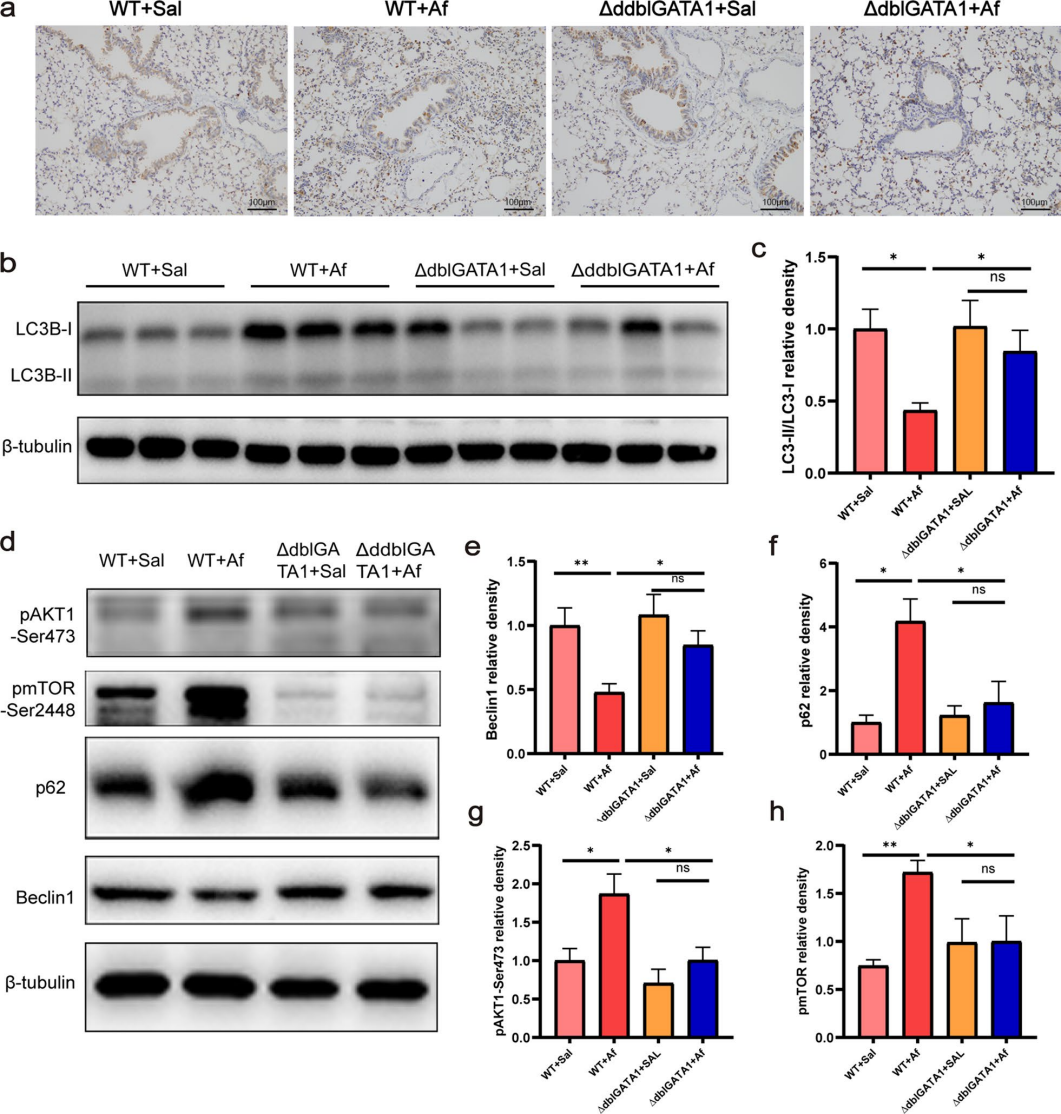
Eosinophils deficiency partially restored the balance of lung autophagy in Af-exposed mice. **a** Representative immunohistochemical images of p62 in the lungs of four groups (magnification: 100×). **b**, **c** The proteins levels of LC3-II and LC3-I were analyzed by Western blots and (**c**) LC3-II/LC3-I ratio were calculated by Image J. **d**–**h** The proteins expressions of p62, beclin1, p-Akt1-ser473, and p-mTOR-ser2448 were analyzed by Western blots and their relative density normalized to β-tubulin. Data are mean ± SEM. (n = 4–6). *p < 0.05, **p < 0.01, and ns = no significance

Previous studies showed autophagy played protective and detrimental effects in allergic lung inflammation [20–22]. Eosinophil deficiency was found to alleviate the Af-induced allergic airway inflammation in the study, then we further assessed how autophagy responded to eosinophil deficiency in this animal model. The conversion of LC3B-I to LC3B-II represents the level of autophagy flux, and p62 is the transport machine of cargo [27]. As shown in Fig. 5a, p62 was highly expressed in lungs of Af-exposed wild-type mice, and no evident change was observed in Af-exposed ∆dblGATA1 mice relative to the saline controls. Western blot analysis showed that Af-exposed wild-type mice had reduced levels of lung LC3B-II/LC3B-I (Fig. 5b, c) and beclin1 (Fig. 5d, e) and increased level of p62 (Fig. 5d, f), all of which were reversed partially in ∆dblGATA1 mice with Af challenge (p < 0.05). Additionally, levels of p-Akt1 and p-mTOR of lungs were increased in the wild-type mice with Af challenge (Fig. 5d, g, h), suggesting the classical Akt/mTOR autophagy pathway was activated, which was not observed in two groups of ∆dblGATA1 mice.

### The analysis of lung transcriptome in ∆dblGATA1 mice after Af sensitization and challenge

**Fig. 6:**
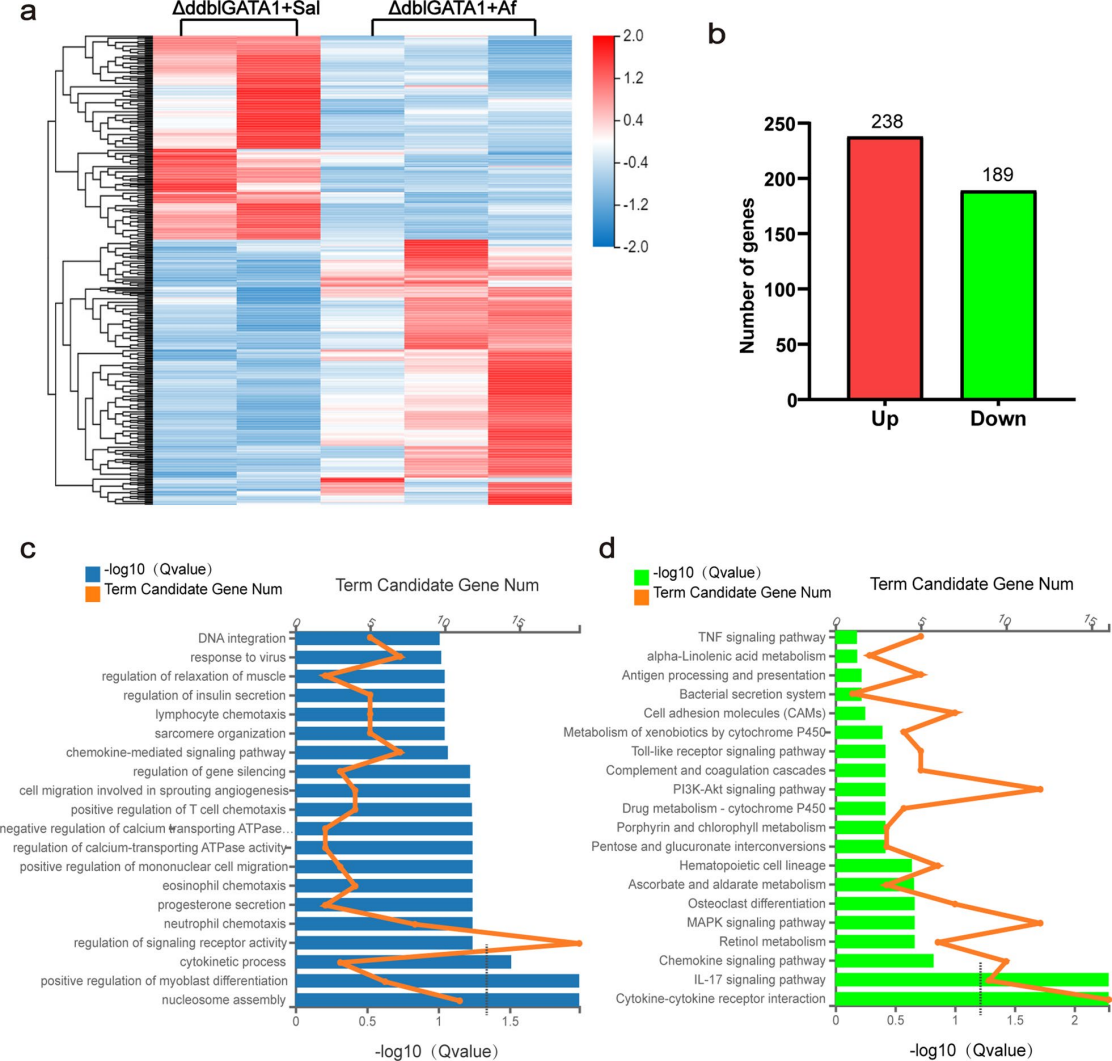
Af-induced transcriptome in ∆dblGATA1 mice. **a** RNA-Seq heat map for the lungs of ∆dblGATA1 mice challenged with saline or Af. **b** Summary of the numbers of differentially expressed genes (DEG). **c** Gene Ontology (GO) analysis of DEGs showing the top 20 enriched biological processes (Q value < 0.05). **d** KEGG analysis of DEGs showing the top 20 enriched pathways

We next examined the impacts of Af on the whole lung transcriptome in ∆dblGATA1. The heatmap (Fig. 6a, b) showed the distribution of 427 differentially expressed genes (DEGs), consisting of 238 genes upregulated and 189 genes downregulated. The results of GO analysis (Fig. 6c) showed that biological processes linked with these DEGs were mainly the cytokinetic process, positive regulation of myoblast differentiation, and nucleosome assembly. Intriguingly, processes related to chemotaxis of lymphocytes, neutrophils, and eosinophils were enriched but without significance (p > 0.05), which maybe imply a not robust enough initiation of allergic inflammation in the Af-exposed ∆dblGATA1 mice. Besides, IL-17 signaling pathway and cytokine–cytokine receptor interaction were the two dominantly enriched pathways with significance revealed by KEGG analysis (Fig. 6d).

## Discussion

This study showed that compared with wild type mice, Af-exposed ∆dblGATA1 mice demonstrated alleviated airway inflammation, reduced Th2 immune response and mucin production, mitigated pathological process of ER stress and apoptosis, and partially enhanced lung autophagy. We firstly presented that increased apoptosis and reduced autophagy in Af-induced allergic lung inflammation. Additionally, we found that the activated Akt/mTOR pathway in the model was inhibited when eosinophils were deficient. These findings suggest that eosinophils have a profound role in the pathogenesis of Af-induced allergic lung inflammation, and eosinophil deficiency might protect against fungal pulmonary diseases.

In the last two decades, congenitally eosinophil deficient mice such as PHIL and ∆dblGATA1 were employed to investigate the role eosinophils play in airway allergic disorders. Although these studies had some contradictory conclusions due to the background of mice [8], allergens such as HDM [28], or OVA [10], they indeed showed that eosinophils were essential for the recruitment of T lymphocytes and dendritic cells. Our study consistently found that eosinophil deficiency caused damage in type 2 cytokines production after challenged by *Aspergillus fumigatus* allergen [24]. We also identified that mucus secretion was suppressed simultaneously, which might be related to the lower level of IL-13 due to its crucial role in controlling mucus production [29]. In addition, the serum IgE level still kept high level in Af-exposed ∆dblGATA1 mice, indicating eosinophil deficiency did not influence serum IgE production. One possible explanation is that eosinophils are not essential for the maintenance of murine plasma cells in bone marrow [30] or respiratory tract [31] upon immunization. However, IL-4 is the major cytokine driving IgE synthesis by plasma cells [29]. That low level of IL-4 and high serum IgE level coexisted in Af-exposed ∆dblGATA1 mice might require further exploration.

We found a marked increase of neutrophils in BAL of Af-exposed ∆dblGATA1 mice. One study demonstrated that eosinophil ablation during airway challenge could lead to a predominantly neutrophilic steroid-resistant phenotype that was reversible upon the restoration of peripheral eosinophils [32]. These neutrophils were speculated as granule-less eosinophils because of their nuclear morphology of murine eosinophils [24]. Importantly, IL-17 plays a key role in recruiting neutrophils to the inflammatory site [33]. Herein RNA-Seq results showed that the IL-17 signaling pathway was significantly enriched with upregulated cytokines such as CXLC3, TNF, MMP3 (data are not shown) in Af-exposed ∆dblGATA1 mice. The transversion of the primary inflammatory cell from eosinophils to neutrophils warrants further investigation into its underlying mechanism and response to steroid.

Eosinophils are thought to be important for maintaining tissue homeostasis in allergic diseases [6, 34]. We examined whether eosinophil deficiency influenced ER stress, apoptosis, and autophagy in Af-exposed allergic pulmonary inflammation. ER stress has been studied extensively in many diseases such as obesity, diabetes, neurodegenerative diseases, pulmonary fibrosis, and asthma [35]. Here we demonstrated that ER stress and UPR were activated significantly in Af-exposed allergic lung inflammation. One study showed that 4-PBA (an ER stress inhibitor) or phosphoinositide 3-kinase-δ inhibitor IC87114 could dramatically reduce Af-induced UPR and airway inflammation, suggesting that ER stress and PI3K-δ pathway were involved in the allergic lung inflammation [13]. Moreover, ORMDL3 is correlated with the degree of ER stress, whose deficiency protected mice from developing Alternaria-induced allergic airway inflammation [35, 36], implying that ER stress inhibition may provide benefits for fungal allergen-induced pulmonary disorders [14, 15]. Our findings found that eosinophil-deficient mice with Af exposure had a lesser degree of ER stress. Thereby we supposed that eosinophils might be a vital part of regulating the fungal-induced allergic lung stress response.

A few studies described the epithelial apoptosis induced by OVA or HDM in asthmatic murine models, and administration of a broad-spectrum caspase inhibitor could attenuate allergic inflammation [16, 17, 23]. Moreover, the selective induction of eosinophil death also helped resolve the inflammation and restore tissue homeostasis by Bcl-2 inhibitors [34]. It has been shown that bronchial epithelial cells are relatively apoptosis-resistant in contrast to immune cells and distal airway epithelial cells as the responses to Fas (cell-surface death receptor) ligation become more pronounced from proximal to distal epithelium [23, 37]. However, what types of cells occurred apoptosis in our study remains to be explored. Remarkably, although the apoptosis was inhibited in eosinophils-deficient mice after challenge, it is unknown that which type or types of cells was affected and what effects the reduced apoptosis has on the allergic inflammation.

At present, no study has investigated the role autophagy play in Af-induced allergic inflammation. Previous studies showed that autophagy could exert detrimental and beneficial effects on allergic inflammation in asthmatic patients and mouse models [20–22]. Our results firstly demonstrated that lung autophagy was inhibited in Af-exposed allergic mouse models, and we speculated that autophagy may be protective in Af-induced allergic lung inflammation. The decreased autophagy was partially restored under eosinophil deficiency, and we supposed that it might be related with the alleviated inflammatory condition [19]. The close relationship and interplay among ER stress, apoptosis, and autophagy have been reviewed in detail [38], and their internal causality in Af-induced allergic lung inflammation needs to be further studied.

Targeting eosinophil is likely to be effective and promising in controlling fungus related allergic pulmonary diseases. Recently, the monoclonal antibody against IgE (Omalizumab) or IL-5 (Mepolizumab), have shown apparent benefits in patients with SAFS [39, 40] or ABPA [41] in clinical trials. Our findings provided some supporting evidence and potential mechanisms for anti-eosinophils therapy in fungus-induced allergic lung inflammation.

## Limitations

There are some issues to be addressed. First, the mechanism by which Th2 pulmonary immune response, ER stress, apoptosis, and autophagy of lungs were affected under eosinophil deficiency is not profoundly investigated. Second, we did not conduct the transmission electron microscope (TEM) to evaluate autophagy because TEM is the golden standard for autophagy. Third, rescue experiments through the restoration of peripheral eosinophils may be necessary to find the role eosinophils have in Af-exposed allergic lung inflammation. Last, the sample size of RNA seq analysis was only five as well as lacking RNA seq data of wild-type mice, all of which may reduce the efficacy of results.

## Conclusions

Our data suggest that eosinophils play an indispensable role in the pathogenesis of Af-exposed allergic lung inflammation as impaired Th2 immune response, improved airway inflammation, downregulated ER stress and apoptosis, and reverse of reduced autophagic level were observed in eosinophil deficient mice, suggesting that anti-eosinophils therapy may offer promising options for fungal-induced allergic pulmonary diseases.

## Data Availability

The datasets used and analyzed during the current study are available from the corresponding author on reasonable request.

## Abbreviations

Af: *Aspergillus* *fumigatus*
ABPA: Allergic bronchopulmonary aspergillosis
SAFS: Severe asthma with fungal sensitization
BAL: Bronchoalveolar lavage
BALF: Bronchoalveolar lavage fluid
RT-PCR: Real time polymerase chain reaction
ELISA: Enzyme linked immunosorbent assay
ER: Endoplasmic reticulum
UPR: Unfolded protein response
TUNEL: Terminal deoxynucleotidyl transferase-mediated nick end labeling assay
CHOP: C/EBP-homologous protein
GRP78: Glucose-regulated protein 78
ATF4: Activating transcription factor 4
DEG: Differentially expressed gene
